# Supplementary material for: Characterization of two O-methyltransferases involved in the biosynthesis of O-methylated catechins in tea plant
Source: Nat Commun. 2023 Aug 21;14:5075. doi: 10.1038/s41467-023-40868-9 (PMC10442441; doi:10.1038/s41467-023-40868-9)
Supplement: Supplementary file 1 — Supplementary Information [file 41467_2023_40868_MOESM1_ESM.pdf]

Characterization of two *O*-methyltransferases involved in synthesis of *O*-methylated catechins in tea plant

Ji-Qiang Jin[1#], Fu-Rong Qu[1#], Huisi Huang[2#], Qing-Shuai Liu[1#], Meng-Yuan Wei[1#], Yuee Zhou[2], Ke-Lin Huang[3], Zhibo Cui[2], Jie-Dan Chen[1], Wei-Dong Dai[1], Li Zhu[1], Ming-Zhe Yao[1]*, Zhi-Min Zhang[2]*, Liang Chen[1]*

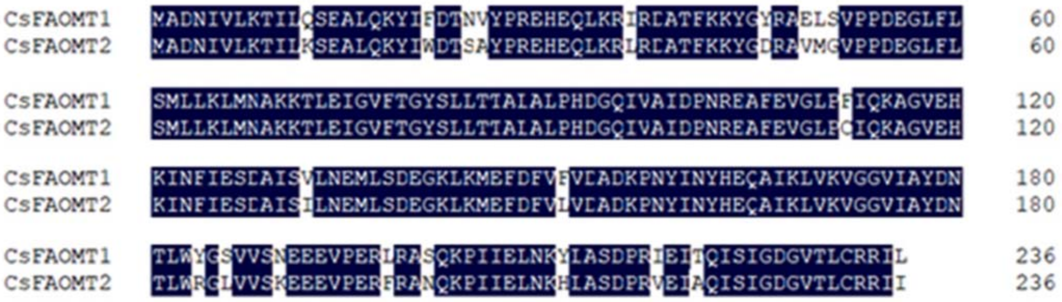

Supplementary Figure 1. Sequence alignment of of CsFAOMT1 and CsFAOMT2.

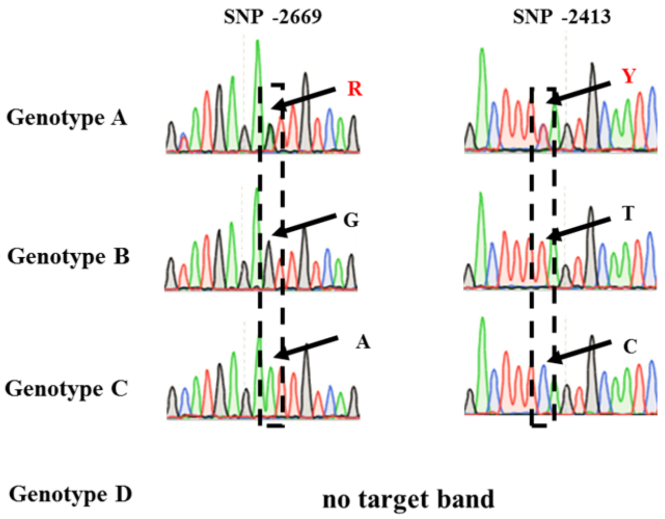

Supplementary Figure 2. Genotyping of *CsFAOMT1* in the F_1 population.

a

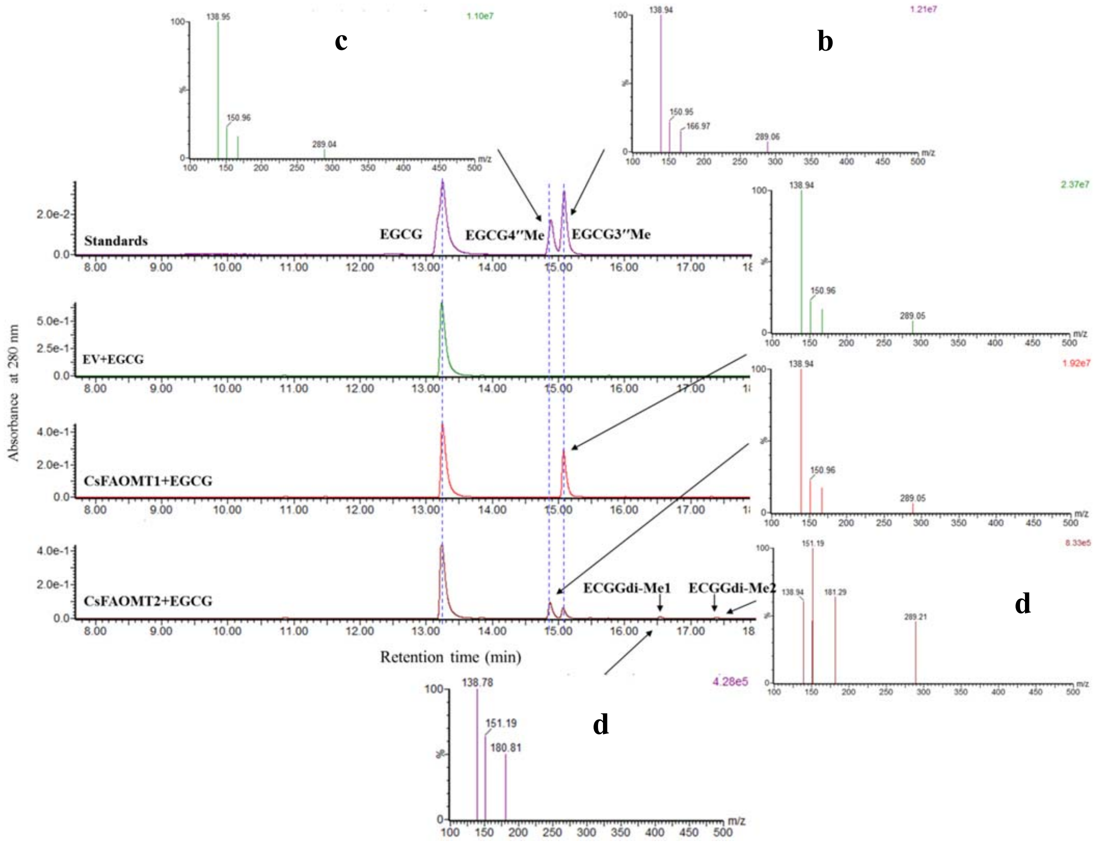

c
b
d
d
Standards
EGCG
EGCG4″Me
EGCG3″Me
EV+EGCG
CsFAOMT1+EGCG
CsFAOMT2+EGCG
ECGGdi-Me1
ECGGdi-Me2
Absorbance at 280 nm
Retention time (min)
m/z

b

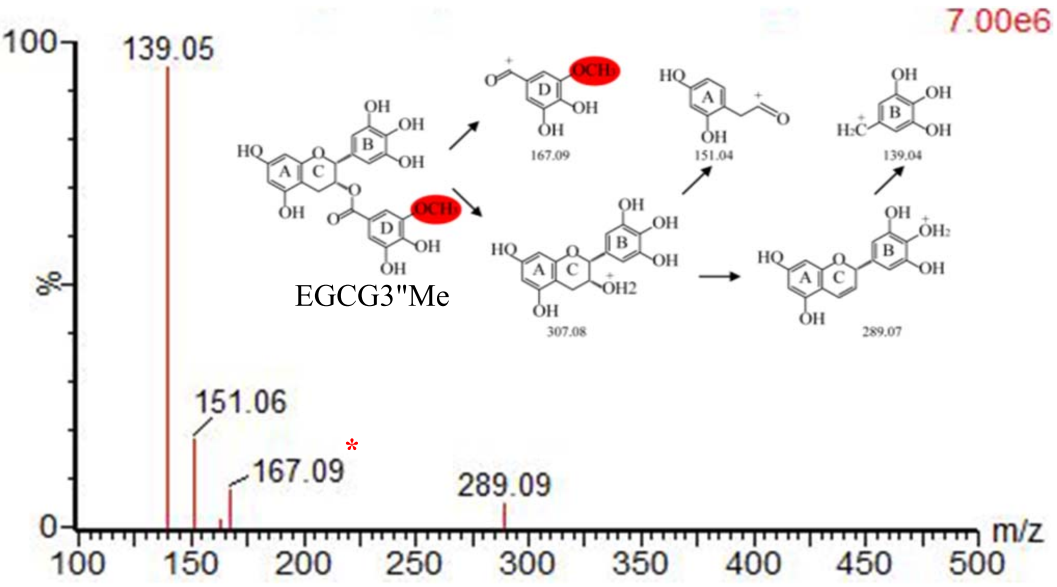

7.00e6
139.05
151.06
167.09
289.09
%
m/z
EGCG3"Me
167.09
151.04
139.04
307.08
289.07

c

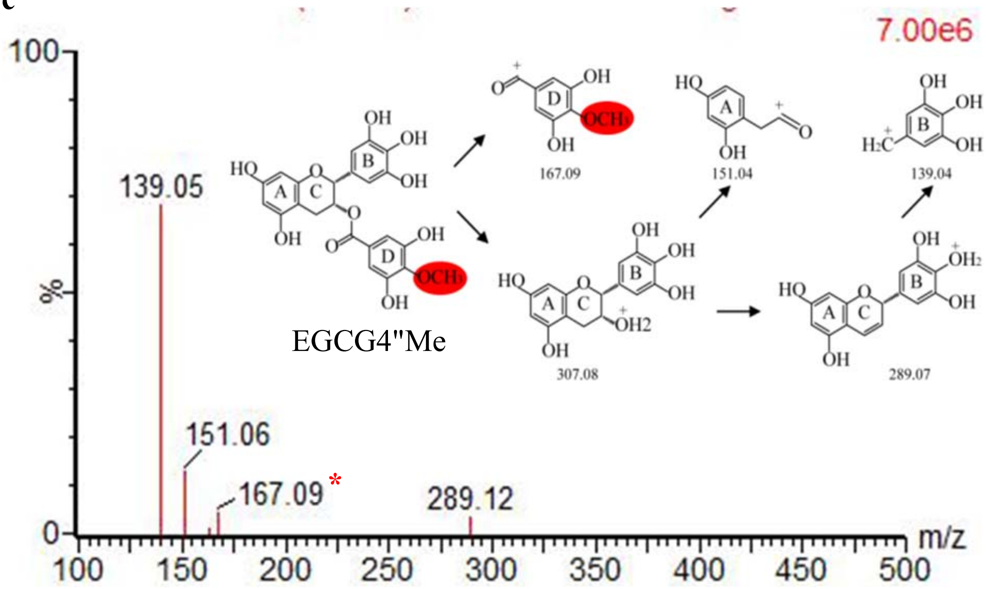
7.00e6
100
%
0
139.05
151.06
167.09 *
289.12
100 150 200 250 300 350 400 450 500
m/z
EGCG4"Me
167.09
151.04
139.04
307.08
289.07

d

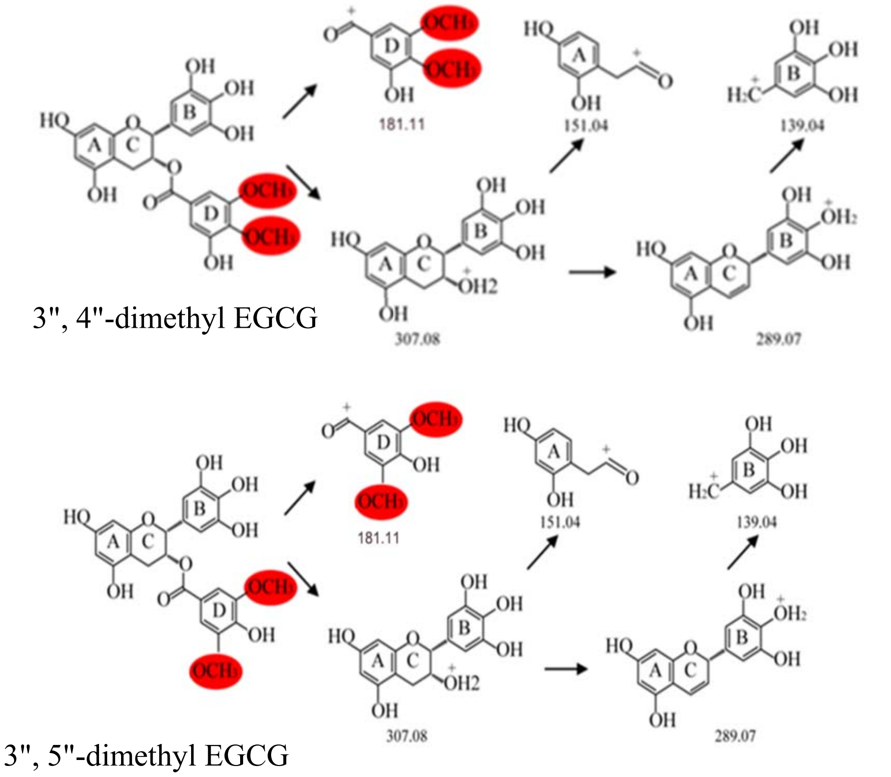
3", 4"-dimethyl EGCG
181.11
151.04
139.04
307.08
289.07
3", 5"-dimethyl EGCG
181.11
151.04
139.04
307.08
289.07

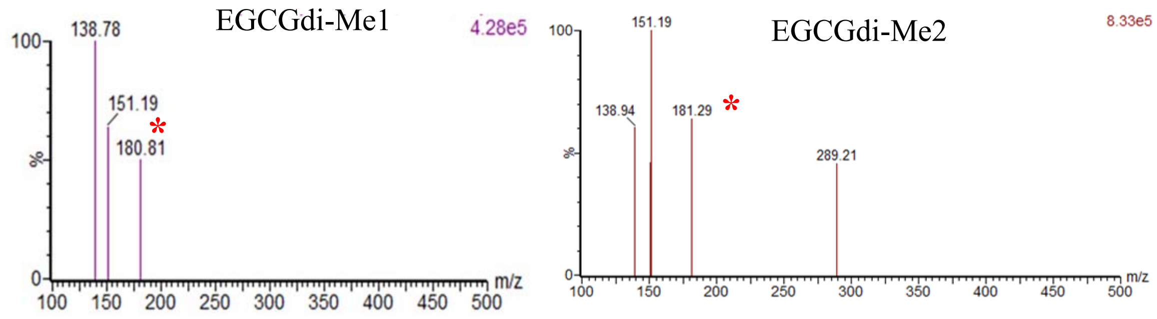

Supplementary Figure 3. Chromatogram and MS^2 fragment ions of the reaction products of *in vitro O*-methyltransferase activity analysis and their authentic compounds. a) *In vitro* reaction products of recombinant CsFAOMT1 and CsFAOMT2 using EGCG as substrate and empty vector (EV) as the control. The details of MS^2 fragment ions of EGCG3″Me, EGCG4″Me, and EGCGdi-Me in the ESI$^+$ mode are presented in **b**), **c**), and **d**) respectively. The fragment containing methyl group on the galloyl group is labeled by a red star. The reaction products were analyzed by UPLC-MS, with the absorbance wavelength set at 280 nm.

a

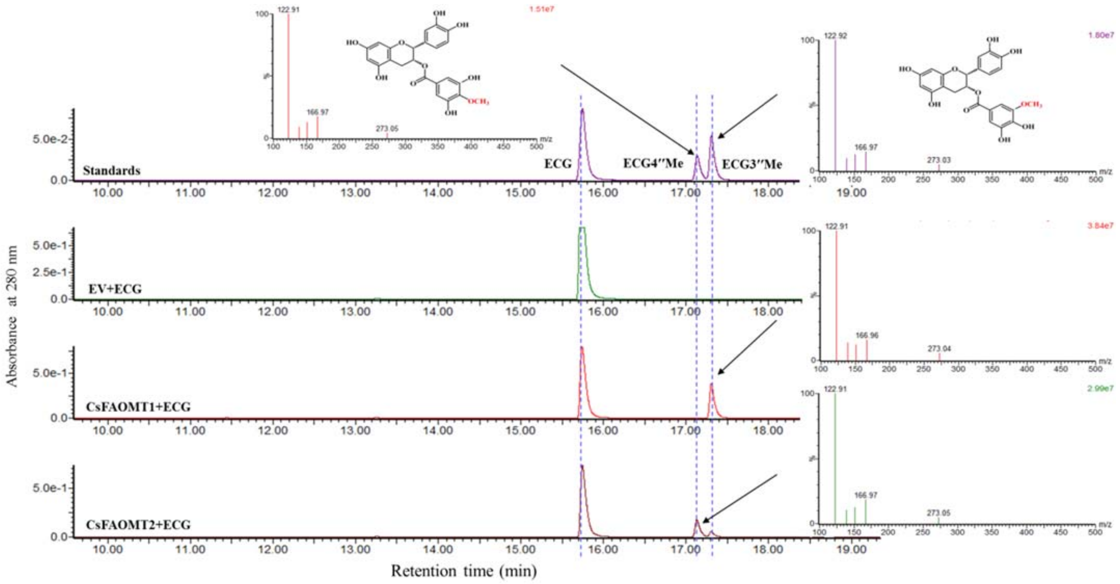

Standards
EV+ECG
CsFAOMT1+ECG
CsFAOMT2+ECG
ECG
ECG4″Me
ECG3″Me
Absorbance at 280 nm
Retention time (min)

b

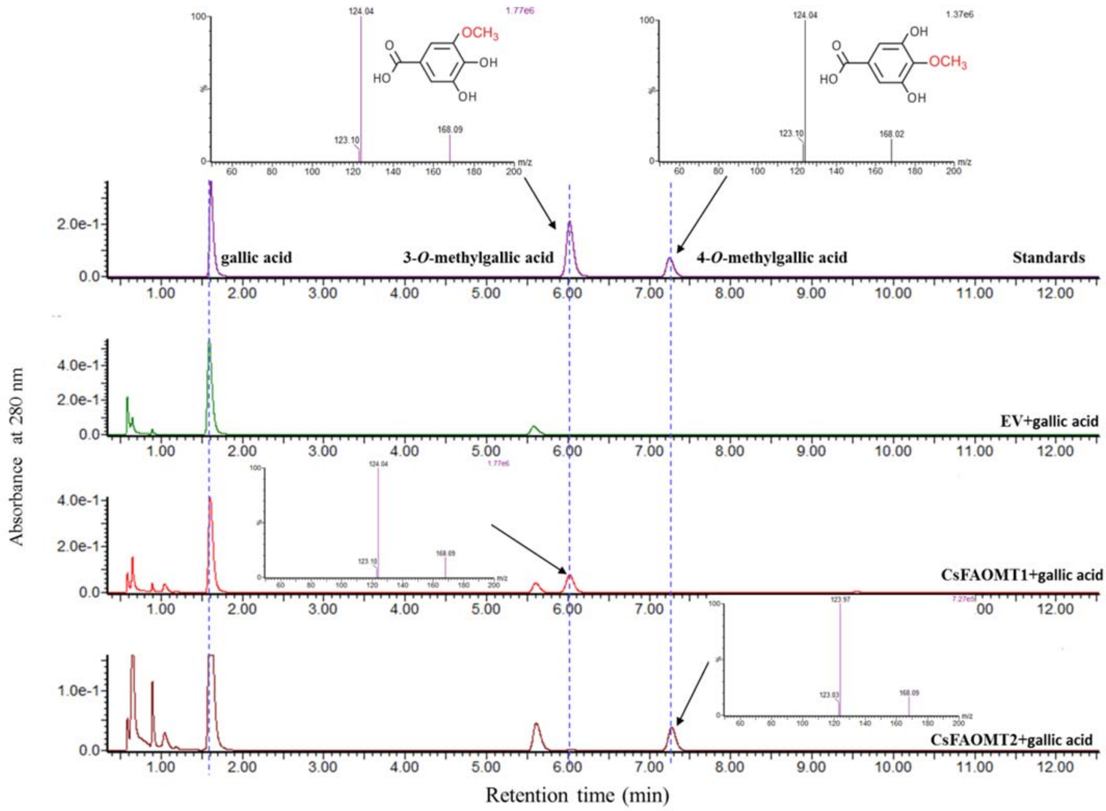

gallic acid
3-*O*-methylgallic acid
4-*O*-methylgallic acid
Standards
EV+gallic acid
CsFAOMT1+gallic acid
CsFAOMT2+gallic acid
Absorbance at 280 nm
Retention time (min)

c

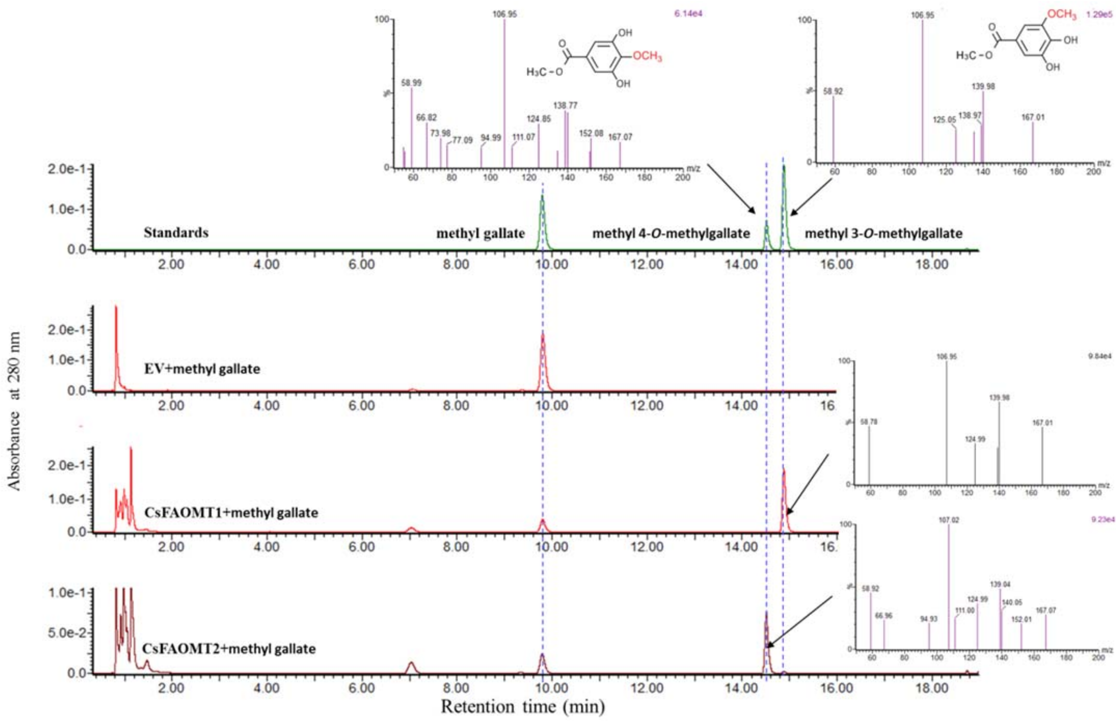

Standards
methyl gallate
methyl 4-O-methylgallate
methyl 3-O-methylgallate
EV+methyl gallate
CsFAOMT1+methyl gallate
CsFAOMT2+methyl gallate
Absorbance at 280 nm
Retention time (min)

d

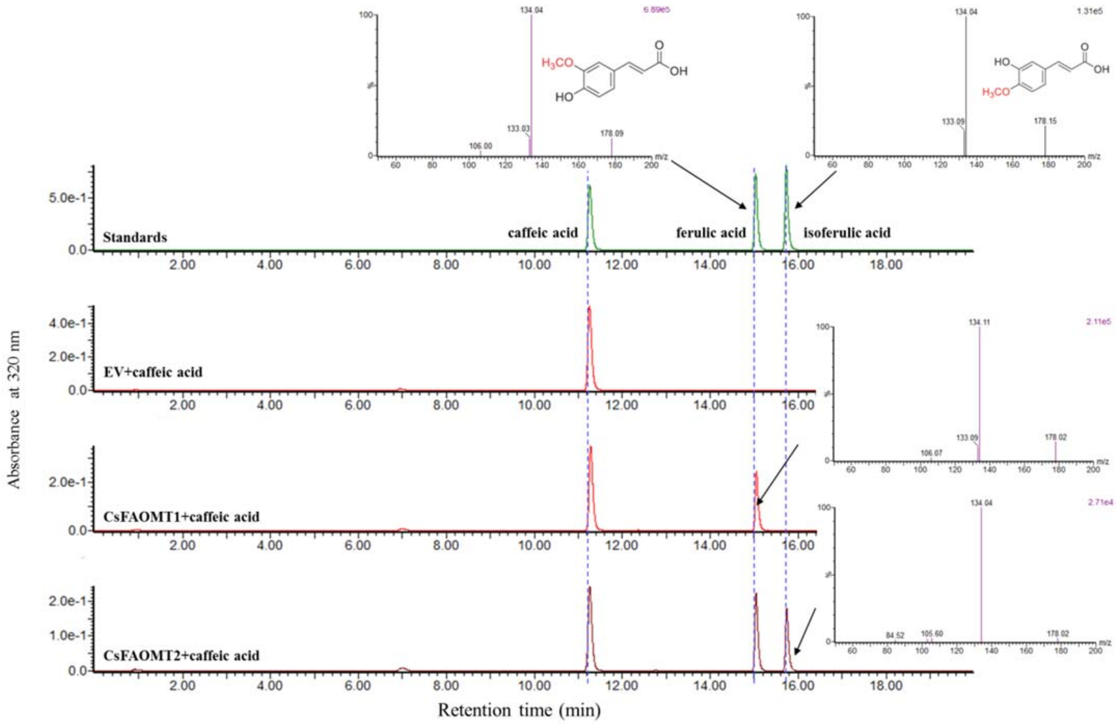

Standards
caffeic acid
ferulic acid
isoferulic acid
EV+caffeic acid
CsFAOMT1+caffeic acid
CsFAOMT2+caffeic acid
Absorbance at 320 nm
Retention time (min)

e

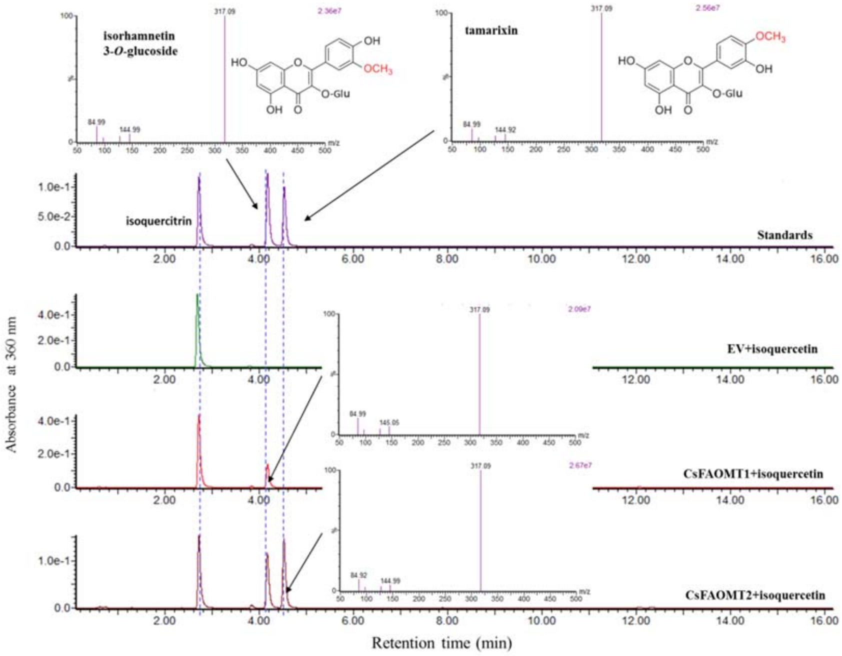

isorhamnetin
3-O-glucoside
tamarixin
isoquercitrin
Standards
EV+isoquercetin
CsFAOMT1+isoquercetin
CsFAOMT2+isoquercetin
Absorbance at 360 nm
Retention time (min)

f

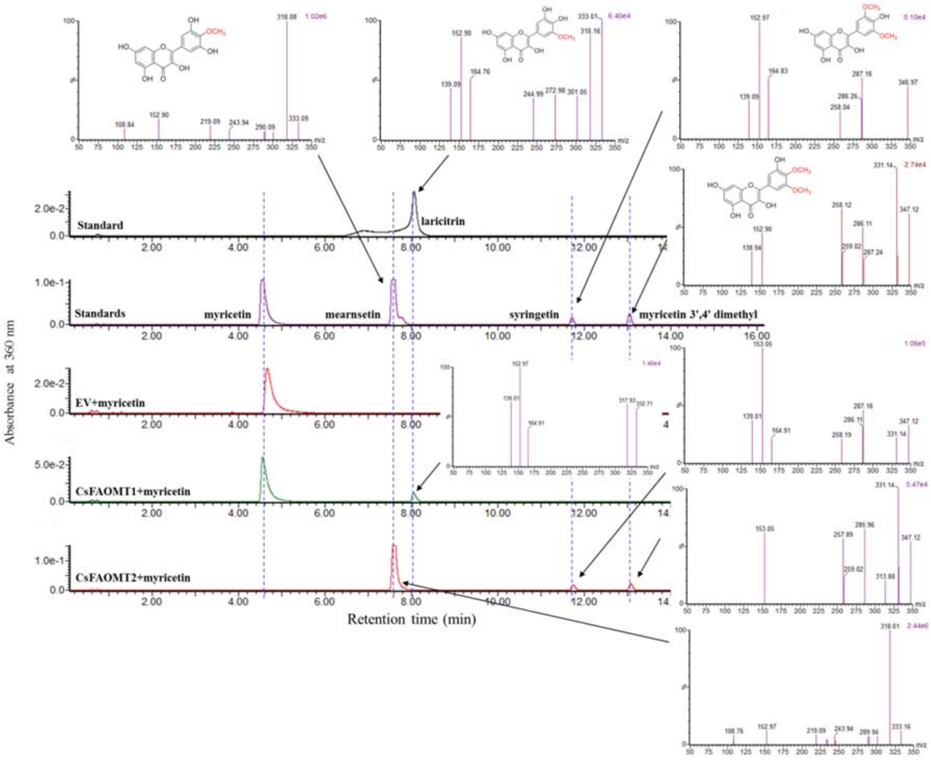

Standard
laricitrin
Standards
myricetin
mearnsetin
syringetin
myricetin 3',4' dimethyl
EV+myricetin
CsFAOMT1+myricetin
CsFAOMT2+myricetin
Absorbance at 360 nm
Retention time (min)

g

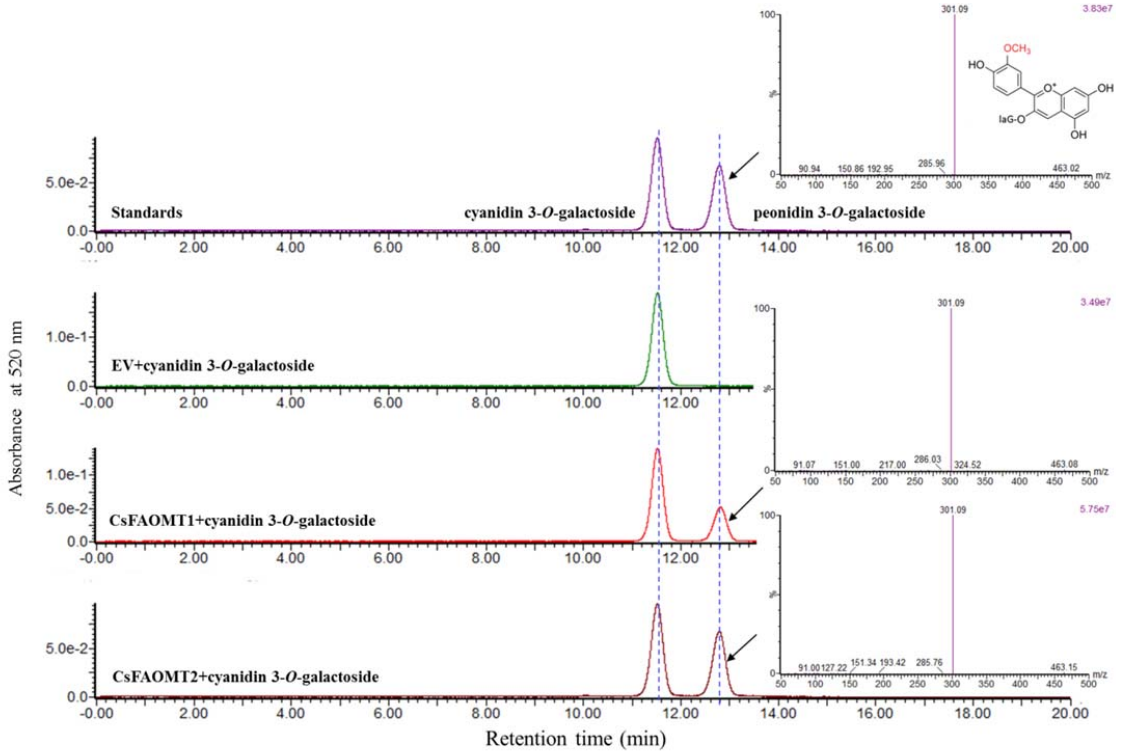

Supplementary Figure 4. Chromatograms and MS^2 fragment ions of the reaction products of *in vitro O*-methyltransferase activity analysis. *In vitro* reaction products of recombinant CsFAOMT1 and CsFAOMT2 using ECG (a, 0.4 mM), gallic acid (b, 0.4 mM), methyl gallate (c, 0.4 mM), caffeic acid (d, 0.2 mM), isoquercitrin (e, 0.2 mM), myricetin (f, 0.2 mM), and cyanidin 3-*O*-galactoside (g, 0.2 mM) as substrate and empty vector (EV) as the control. The details of MS^2 fragment ions of 3-*O*-methylgallic acid, 4-*O*-methylgallic acid, ferulic acid, and isoferulic acid in the ESI$^-$ mode, the other compounds in the ESI$^+$ mode. glu: glucoside, gal: galactoside. The reaction products were analyzed by UPLC-MS.

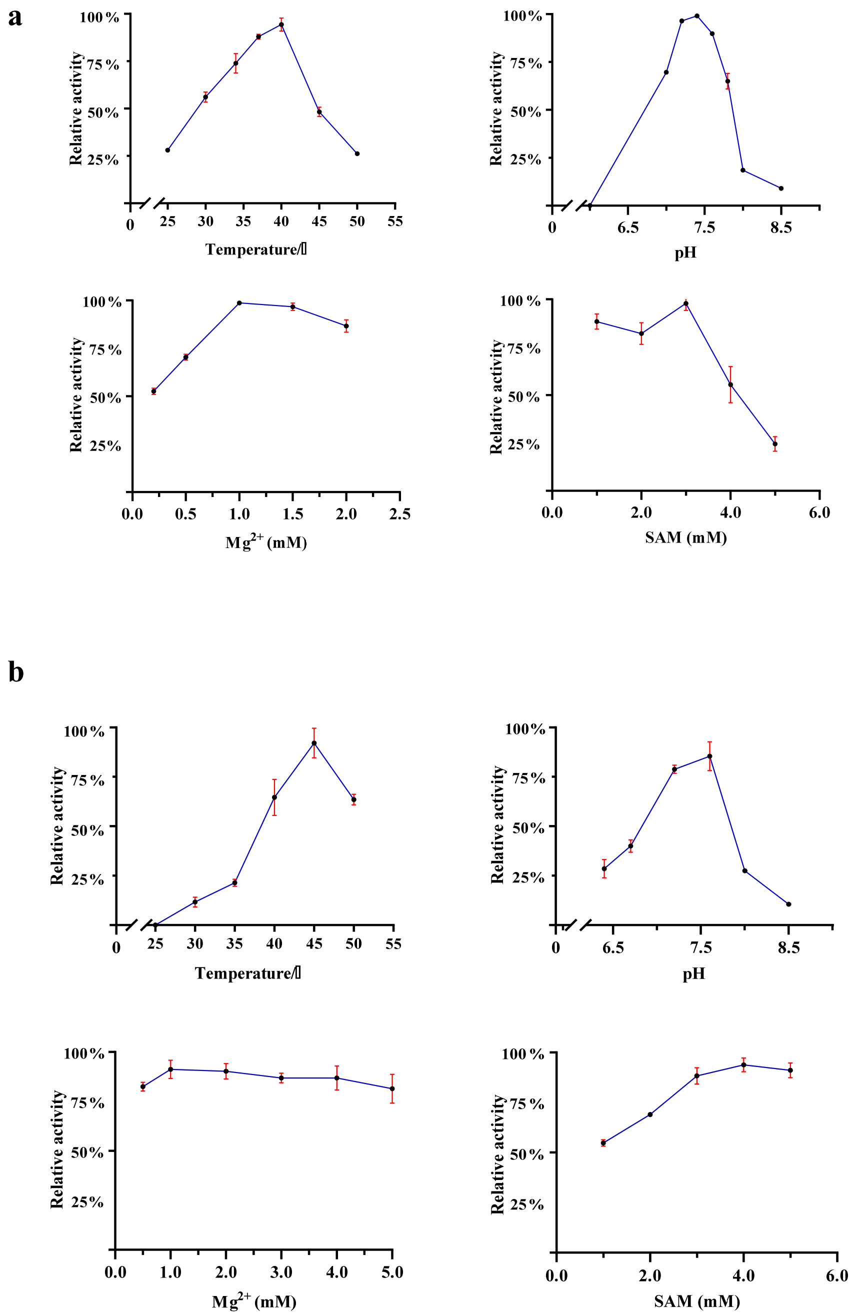

Supplementary Figure 5. Optimal conditions in the enzymatic reaction of CsFAOMT1 (a) and CsFAOMT2 (b). Data in the figure are the mean ± standard deviation of three replicates.

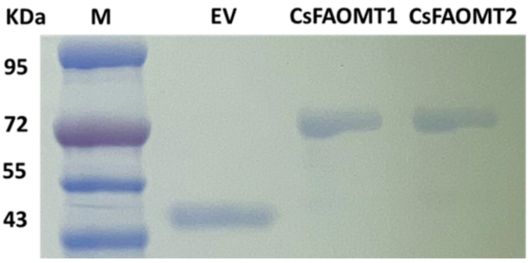

Supplementary Figure 6. SDS-PAGE analysis of the recombinant proteins of empty vector (EV), CsFAOMT1 and CsFAOMT2. The experiment was repeated twice with similar results.

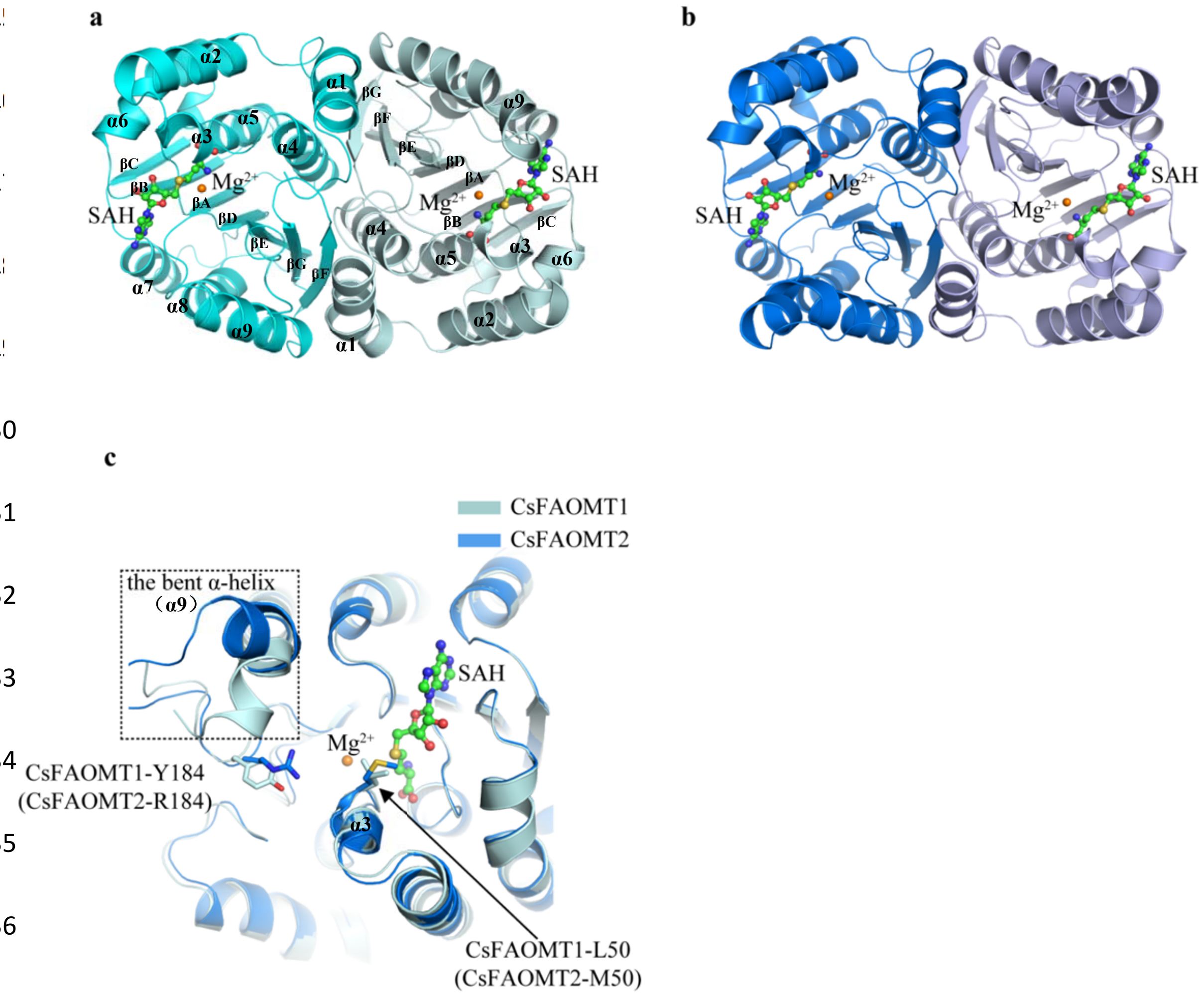

Supplementary Figure 7. Crystal structures of CsFAOMT1 and CsFAOMT2. a) Crystal structure of the CsFAOMT1 dimer. **b)** Crystal structure of the CsFAOMT2 dimer. SAH is shown as a ball-and-stick presentation. Mg^{2+} is shown as brown spheres. **c)** Structural superposition of CsFAOMT1 and CsFAOMT2 monomer. The side chains of the residues vary between CsFAOMT1 and CsFAOMT2 are shown as sticks.

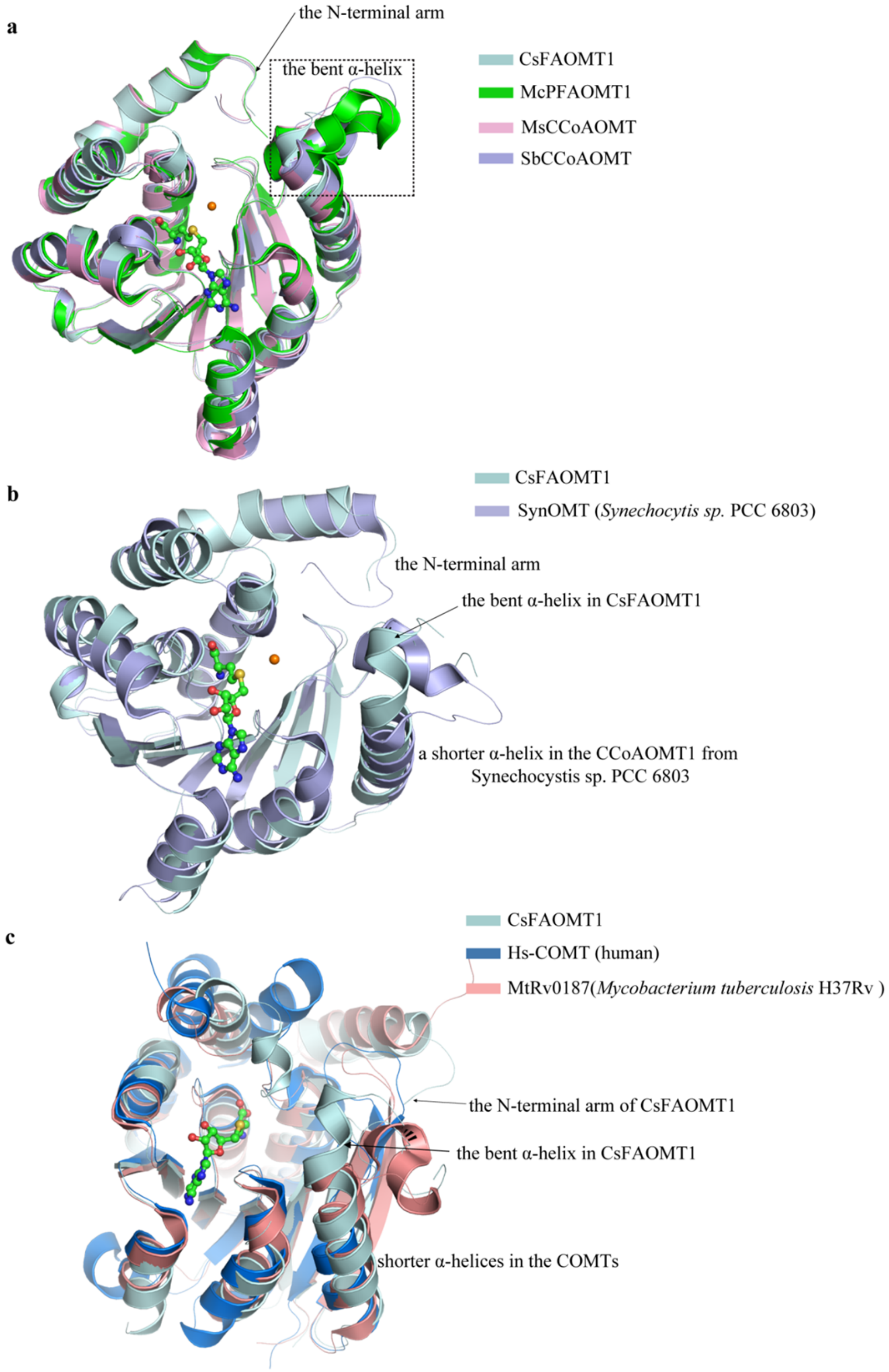
a
the N-terminal arm
the bent α-helix
CsFAOMT1
McPFAOMT1
MsCCoAOMT
SbCCoAOMT
b
CsFAOMT1
SynOMT (Synechocytis sp. PCC 6803)
the N-terminal arm
the bent α-helix in CsFAOMT1
a shorter α-helix in the CCoAOMT1 from Synechocystis sp. PCC 6803
c
CsFAOMT1
Hs-COMT (human)
MtRv0187(Mycobacterium tuberculosis H37Rv)
the N-terminal arm of CsFAOMT1
the bent α-helix in CsFAOMT1
shorter α-helices in the COMTs

Supplementary Figure 8. Structural superposition of CsFAOMT1 with other cation-dependent OMTs. a) Structural superposition of CsFAOMT1 with other plant O-methyltransferases: CCoACOMT from *Medicago sativa* (MsCCoACOMT, PDB code 1SUI), McPFAOMT from *Mesembryanthemum crystallinum* (PDB code 3C3Y) and SbCCoACOMT from Sorghum bicolor (PDB code 5KVA). SHA in CsFAOMT1 is shown as a ball-and-stick presentation**.** Mg^{2+} in CsFAOMT1 is shown as brown sphere. **b)** Structural superposition of CsFAOMT1 with CCoACOMT-like from *Synechocytis sp.* PCC 6803 (SynOMT, PDB code 3CBG). **c)** Structural superposition of CsFAOMT1 with COMTs from human (Hs-CMOT, PDB code 6I3D) and *Mycobacterium tuberculosis* H37Rv (MtRv0187, PDB code 6JCL). COMTs do not contain the N-terminal arm.

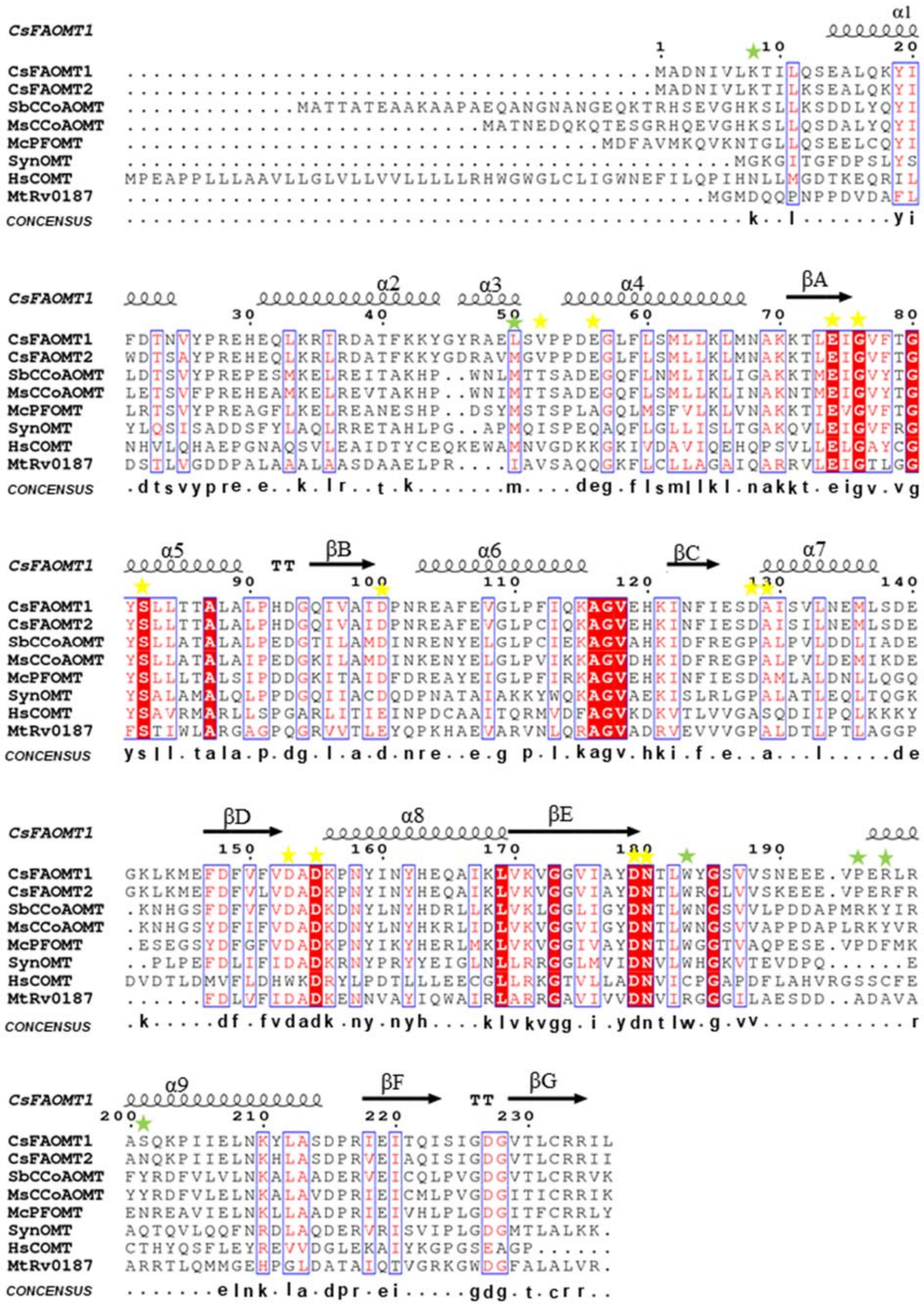

Supplementary Figure 9. Sequence alignment of cation-dependent OMTs. Sequence alignment of CsFAOMTs with the OMTs used for structural alignment in Supplementary Figure 8. The secondary structure of CsFAOMT1 is displayed above and the consensus

sequences (⩾0.8) of the five OMTs from plants are plotted below the alignment. Residues involved in divalent metal ion and cofactor binding are marked by yellow asterisks; residues involved in substrate recognition are marked by green asterisks.

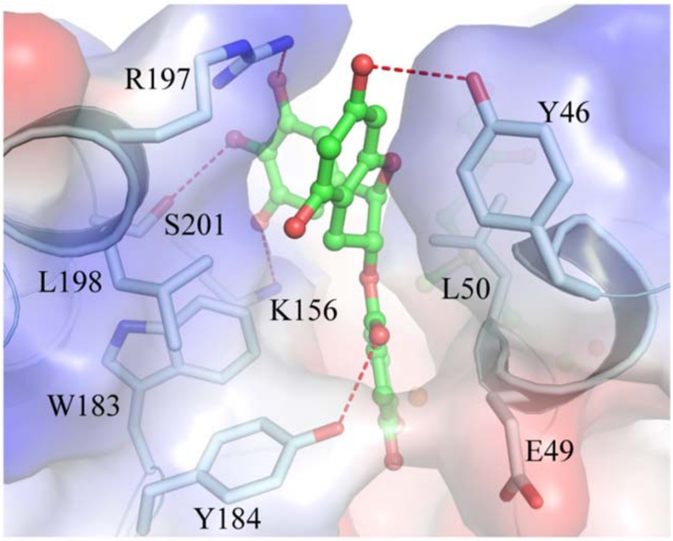

Supplementary Figure 10. Close-up view of the EGCG-binding active site of CsFAOMT1. EGCG was docking into the active site and shown as ball-and-stick presentation. Hydrogen bonds are shown as red dash.

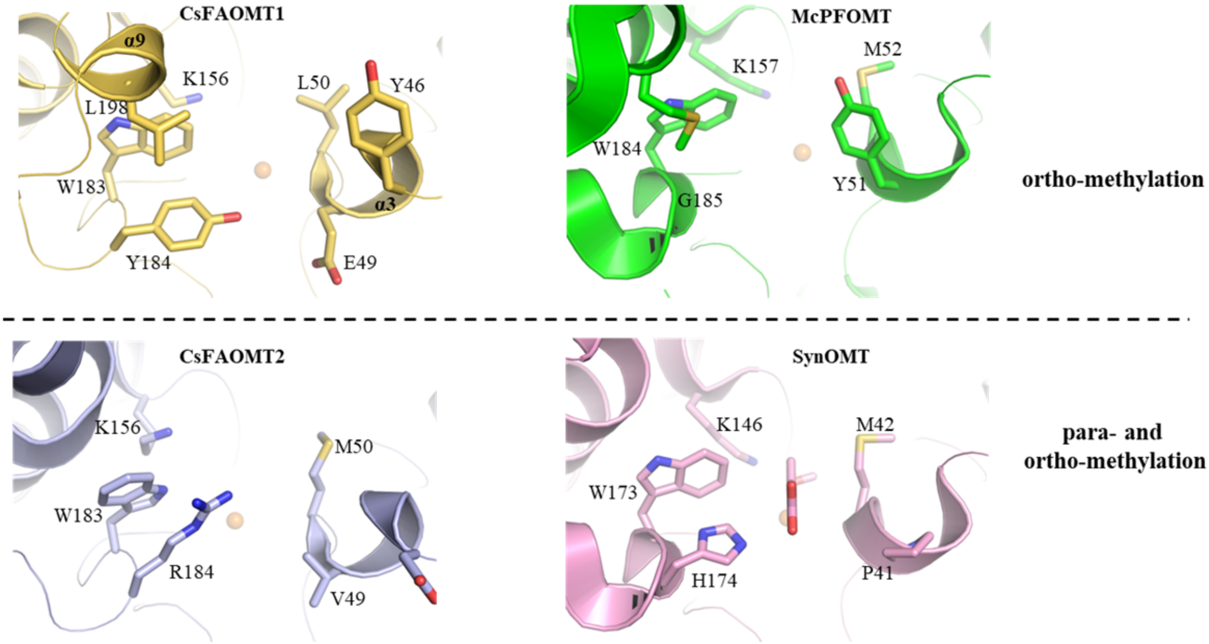

Supplementary Figure 11. Comparison of the active sites of CsFAOMT1, CsFAOMT2, McPFMOT and SynOMT. Mg^{2+} ions are shown as brown sphere.

Supplementary Table 1. Primer sequences used in study.

Name	Primer sequence (5′-3′)	Function
FAOMT-F1	AGTCTYATCTGRGAAAACAT	Amplifying the coding region of both Cs*FAOMT1* and Cs*FAOMT2*
FAOMT-R1	GATAACAACATTAYAAACGG	
FAOMT-F2	GTTGTTTAGTCTYATCTGRGA	
FAOMT-R2	GACTTAAAATCCATGGTAAC	
SNP-F	TCTGAATAGTAAACTGCAAC	Genotyping of *CsFAOMT1*
SNP-R	CTTGTTGGGGGAAGGTTTAC	
18S-F	TCTCAACCATAAACGATGCCGACCAG	qRT-PCR
18S-R	TTTCAGCCTTGCGACCATACTCCC	
FAOMT1-RT-F	TATTCTCTTCTTACCACTGCCCTT	
FAOMT1-RT-R	CTCCATTTTCAACTTACCCTCATC	
FAOMT2-RT-F	GGTAAAAGTAGGAGGAGTTATTGCG	
FAOMT2-RT-R	TAGATTATGCGTCTGCACAGTGTGA	

Supplementary Table 2. Contents of major catechin components in different tissues

Tissues	ECG (mg g^{-1})	EGCG (mg g^{-1})	EC (mg g^{-1})	EGC (mg g^{-1})
Young leaves	17.53 ± 0.97a	92.86 ± 2.94a	8.73 ± 0.07a	32.80 ± 0.42a
Mature leaves	6.92 ± 0.81c	40.86 ± 1.63b	6.76 ± 0.85b	33.74 ± 2.65a
Old leaves	1.56 ± 0.70d	13.29 ± 2.76d	3.99 ± 0.19cd	19.26 ± 3.12b
Flower buds	10.84 ± 1.14b	19.62 ± 0.54c	5.13 ± 0.54c	5.45 ± 0.27c
Flowers	6.03 ± 0.25c	13.25 ± 0.71d	3.51 ± 0.16d	3.40 ± 0.22cd
Seeds	0.01 ± 0.02d	0.03 ± 0.03e	0.93 ± 0.15e	0.02 ± 0.00d
Roots	0.00 ± 0.00d	0.00 ± 0.00e	1.76 ± 0.34e	0.00 ± 0.00d
P	1.86×10^{-13}	5.36×10^{-18}	1.85×10^{-11}	1.01×10^{-13}

Data in the table are the mean ± standard deviation of three samples. The experiment was repeated twice. Different letters following the values indicate significant differences between tissues using one-way ANOVA at $P < 0.05$.

Supplementary Table 3. Compounds identified by LC-MS analysis of reaction products produced by recombinant CsFAOMT1 and CsFAOMT2

Substrate	Retention time (min)	$[M+H]^+$ m/z	Product	Retention time (min)	$[M+H]^+$ m/z	Methylated position
EGCG	13.3	459	EGCG4″Me	14.9	473	4″
			EGCG3″Me	15.1	473	3″
			EGCG3-diMe1	16.6	487	unknown
			EGCG3-diMe2	17.5	487	unknown
ECG	15.7	443	ECG4″Me	17.1	457	4″
			ECG3″Me	17.3	457	3″
Gallic acid	1.7	169*	3-*O*-methylgallic acid	6.1	183*	3
			4-*O*-methylgallic acid	7.3	183*	4
Methyl gallate	9.8	185	Methyl 4-*O*-methylgallate	14.5	199	4
			Methyl 3-*O*-methylgallate	14.9	199	3
Caffeic acid	11.3	179*	Ferulic acid	15.1	193*	3
			Isoferulic acid	15.8	193*	4
Isoquercitrin	2.7	465	Isorhamnetin 3-*O*-glucoside	4.2	479	3′
			Tamarixin	4.5	479	4′
Myricetin	4.6	319	Mearnsetin	7.6	333	4′
			Laricitrin	8.1	333	3′

			Syringetin	11.7	347	3′, 5′
			Myricetin 3',4' dimethyl ether	13.1	347	3′, 4′
Cyanidin 3-*O*-galactoside	11.5	449	Peonidin 3-*O*-galactoside	12.8	463	3′

$[M+H]^+$, theoretical mass (m/z) of the compound in positive mode; *, m/z values indicated with an asterisk represent $[M-H]^-$ values because they have been determined in negative mode.

Supplementary Table 4. Enzyme Kinetics of CsFAOMT1 and CsFAOMT2.

Substrate	Protein	K_m (μM)	*Vmax* (nmol mg^{-1} min^{-1})	K_{cat} (×10^{-3} S^{-1})	K_{cat}/K_m (M^{-1} S^{-1})	R^2
EGCG	CsFAOMT1	95.79 ± 7.23	311.07 ± 19.83	366.93 ± 23.39	3830.57	0.9732
	CsFAOMT2	20.88 ± 1.73	119.97 ± 4.52	141.20 ± 5.36	6762.45	0.9259
ECG	CsFAOMT1	13.54 ± 0.64	215.07 ± 3.08	253.69 ± 3.64	18736.33	0.9631
	CsFAOMT2	43.91 ± 4.07	212.63 ± 210.61	250.30 ± 13.82	5700.30	0.9541
Gallic acid	CsFAOMT1	214.67 ± 11.43	271.67 ± 12.26	320.46 ± 14.46	1492.80	0.9877
	CsFAOMT2	64.93 ± 6.14	199.20 ± 12.35	234.49 ± 14.54	3611.43	0.9611
Methyl gallate	CsFAOMT1	3.83 ± 0.36	132.27 ± 5.85	156.02 ± 6.90	40736.29	0.9548
	CsFAOMT2	54.52 ± 12.64	357.23 ± 22.17	420.52 ± 26.10	7713.13	0.9497
Caffeic acid	CsFAOMT1	130.77 ± 7.22	160.10 ± 4.65	188.85 ± 5.49	1444.14	0.9884
	CsFAOMT2	144.50 ± 10.95	529.6 ± 28.69	623.43 ± 33.77	4314.39	0.9876
Isoquercitrin	CsFAOMT1	2518.00 ± 315.92	481.83 ± 30.94	568.37 ± 36.50	225.72	0.9797
	CsFAOMT2	101.95 ± 18.41	187.70 ± 13.50	220.95 ± 15.89	2167.24	0.9827
Myricetin	CsFAOMT1	3.04 ± 0.71	102.26 ± 17.24	120.63 ± 20.34	39680.92	0.9361
	CsFAOMT2	11.08 ± 0.60	101.29 ± 4.44	119.23 ± 5.23	10760.83	0.9562
Cyanidin 3-*O*-galactoside	CsFAOMT1	87.49 ± 20.73	83.13 ± 13.21	98.06 ± 15.59	1120.81	0.9504
	CsFAOMT2	78.85 ± 17.31	24.25 ± 2.60	28.54 ± 3.06	361.95	0.9560

All enzyme kinetic parameters were determined when the substrate was fully saturated, and the data in the table are the mean ± standard deviation of three replicates.

Supplementary Table 5. Relative activities (%) in different *S*-adenosyl-L-methionine concentrations of the enzymatic reaction of CsFAOMT1.

Substrate	*S*-adenosyl-L-methionine (mM)		
	**0.5**	**1**	**3**
ECG	53.75 ± 1.34	64.17 ± 5.52	98.71 ± 1.13
Gallic acid	72.89 ± 5.41	80.30 ±7.83	95.76 ± 6.20
Methyl gallate	47.23 ± 4.28	48.29 ± 5.61	86.51 ± 12.66
Caffeic acid	69.70 ± 7.25	96.33 ± 3.24	67.01 ± 17.30
Isoquercitrin	83.34 ± 5.28	99.94 ± 0.10	75.00 ± 5.82
Myricetin	42.38 ± 13.53	56.72 ± 2.91	83.75 ± 18.66
Cyanidin 3-*O*-galactoside	77.55 ± 1.89	94.71 ± 5.52	61.74 ± 8.23

Data in the table are the mean ± standard deviation of three replicates.

Supplementary Table 6. Relative activities (%) in different *S*-adenosyl-L-methionine concentrations of the enzymatic reaction of CsFAOMT2.

Substrate	*S*-adenosyl-L-methionine (mM)		
	**0.5**	**2**	**4**
ECG	56.46 ± 1.90	98.79 ± 1.05	87.11 ± 8.78
Gallic acid	51.91 ± 0.69	95.89 ± 4.87	53.80 ± 2.27
Methyl gallate	49.14 ± 1.32	90.24 ± 1.54	95.76 ± 4.17
Caffeic acid	48.93 ± 7.73	87.65 ± 10.93	37.51 ± 1.17
Isoquercitrin	78.27 ± 7.68	96.36 ± 3.27	75.31 ± 1.90
Myricetin	22.89 ± 6.45	73.96 ± 16.49	93.95 ± 5.49
Cyanidin 3-*O*-galactoside	50.89 ± 8.16	86.95 ± 13.50	49.10 ± 16.06

Data in the table are the mean ± standard deviation of three replicates.

Supplementary Table 7. Data collection and refinement statistics

	CsFAOMT1-SAH (8GXO)	CsFAOMT2-SAH (8GXN)
**Data collection**		
Space group	$P3_221$	$P3_121$
Cell dimensions		
a, b, c (Å)	99.899,99.899, 99.03	74.688, 74.688, 81.523
α, β, γ (°)	90.00, 90.00, 120.00	90.00, 90.00, 120.00
Resolution (Å)	65.15 -1.74 (1.83 -1.74)[a]	64.68-1.34(1.42-1.34)
R_{merge}	0.075(1.168)	0.251(0.970)
$I/\sigma(I)$	23.8(2.7)	8.2(2.7)
$CC_{1/2}$	1.000(0.855)	0.987(0.866)
Completeness (%)	90.50(100.00)	100.00(100.00)
Redundancy	19.0(19.8)	19.0(15.5)
**Refinement**		
Resolution (Å)	43.26-1.74	27.54-1.34
No. reflections	53332	58959
R_{work} / R_{free}	0.1856/0.2201	0.1654/0.1717
No. atoms		
Protein	3492	1835
Ligand	52	26
Mg^{2+}	2	1
Water	506	258
B factors		
Protein	27.65	21.27
Ligand	39.65	31.27
Water	39.81	32.40
Mg^{2+}	39.65	31.27
R.m.s.deviations		
Bond lengths (Å)	0.007	0.007
Bond angles (°)	1.02	1.00

[a]Values in parentheses are for highest-resolution shell

Supplementary Table 8. Multiple reaction monitoring transitions, cone voltage, collision energy voltage, and linear range for catechins measurement.

Substrate	Retention time (min)	MRM transitions	Cone voltage (V)	Collision energy (V)	Linear range (μg/mL)	Regression coefficient (r)
EC	13.1	291 > 139	30	20	0.1−12	0.9999
EGC	10.3	307 > 139	30	20	0.1−12	0.9997
ECG	15.8	443 > 139	30	20	0.1−12	0.9999
EGCG	13.3	459 > 139	30	20	0.1−12	0.9998
EGCG3″ Me	15.1	473 > 139	15	20	0.1−12	1
EGCG4″ Me	14.9	473 > 139	15	20	0.1−12	0.9992

Supplementary Table 9. Cone voltage and collision energy voltage for identification of reaction products produced by recombinant CsFAOMT1 and CsFAOMT2 by LC-MS/MS.

Product	Retention time (min)	Cone voltage (V)	CE voltage (V)
EGCG4″Me	14.9	30	15
EGCG3″Me	15.1	30	15
EGCGdiMe1	16.6	30	15
EGCGdiMe2	17.5	30	15
ECG4″Me	17.1	30	15
ECG3″Me	17.3	30	15
3-*O*-methylgallic acid	6.1	10	20
4-*O*-methylgallic acid	7.3	10	20
Methyl 4-*O*-methylgallate	14.5	10	20
Methyl 3-*O*-methylgallate	14.9	10	20
Ferulic acid	15.1	10	20
Isoferulic acid	15.8	10	20
Isorhamnetin 3-*O*-glucoside	4.2	10	20

Tamarixin	4.5	10	20
Mearnsetin	7.6	10	25
Laricitrin	8.1	10	25
Syringetin	11.7	10	25
Myricetin 3',4' dimethyl ether	13.1	10	25
Peonidin 3-*O*-galactoside	12.8	10	20
