# Supplementary material for: Characterization of two O-methyltransferases involved in the biosynthesis of O-methylated catechins in tea plant
Source: Nat Commun. 2023 Aug 21;14:5075. doi: 10.1038/s41467-023-40868-9 (PMC10442441; doi:10.1038/s41467-023-40868-9)
Supplement: Supplementary file 3 — Description of Additional Supplementary Files [file 41467_2023_40868_MOESM3_ESM.pdf]

Description of Additional Supplementary Files:

Supplementary Data 1: Ninety-four up-regulated differentially expressed genes of bulked segregant RNA sequencing (BSR-Seq).

Supplementary Data 2: The correlation between the transcriptional level of the genes and the level of EGCG3"Me in the 27 tea accessions.
